# Towards Efficient and Accurate SARS-CoV-2 Genome Sequence Typing Based on Supervised Learning Approaches

**DOI:** 10.3390/microorganisms10091785

**Published:** 2022-09-04

**Authors:** Miao Miao, Erik De Clercq, Guangdi Li

**Affiliations:** 1 Hunan Provincial Key Laboratory of Clinical Epidemiology, Xiangya School of Public Health, Central South University, Changsha 410078, China; 2 Department of Microbiology, Immunology and Transplantation, Rega Institute for Medical Research, KU Leuven, 3000 Leuven, Belgium; 3 Hunan Children’s Hospital, Changsha 410007, China

**Keywords:** SARS-CoV-2, variants, sequence typing, machine learning, template matching, ensemble

## Abstract

Despite the active development of SARS-CoV-2 surveillance methods (e.g., Nextstrain, GISAID, Pangolin), the global emergence of various SARS-CoV-2 viral lineages that potentially cause antiviral and vaccine failure has driven the need for accurate and efficient SARS-CoV-2 genome sequence classifiers. This study presents an optimized method that accurately identifies the viral lineages of SARS-CoV-2 genome sequences using existing schemes. For Nextstrain and GISAID clades, a template matching-based method is proposed to quantify the differences between viral clades and to play an important role in classification evaluation. Furthermore, to improve the typing accuracy of SARS-CoV-2 genome sequences, an ensemble model that integrates a combination of machine learning-based methods (such as Random Forest and Catboost) with optimized weights is proposed for Nextstrain, Pangolin, and GISAID clades. Cross-validation is applied to optimize the parameters of the machine learning-based method and the weight settings of the ensemble model. To improve the efficiency of the model, in addition to the one-hot encoding method, we have proposed a nucleotide site mutation-based data structure that requires less computational resources and performs better in SARS-CoV-2 genome sequence typing. Based on an accumulated database of >1 million SARS-CoV-2 genome sequences, performance evaluations show that the proposed system has a typing accuracy of 99.879%, 97.732%, and 96.291% for Nextstrain, Pangolin, and GISAID clades, respectively. A single prediction only takes an average of <20 ms on a portable laptop. Overall, this study provides an efficient and accurate SARS-CoV-2 genome sequence typing system that benefits current and future surveillance of SARS-CoV-2 variants.

## 1. Introduction

Severe acute respiratory syndrome coronavirus 2 (SARS-CoV-2), the causative virus of coronavirus disease 2019 (COVID-19), emerged at the end of 2019, burdening both the global economy and public health [1,2,3,4]. Next-generation sequencing has provided an unprecedented opportunity to monitor the COVID-19 pandemic in real-time [5,6]. During the pandemic, vast amounts of SARS-CoV-2 genome sequences have been accumulated at ever-growing rates and shared in the public database. As of 12 July 2022, more than 10 million SARS-CoV-2 genome sequences worldwide are available to researchers in the online database Global Initiative on Sharing all Individual Data (GISAID) [7] (available at https://www.gisaid.org/ (accessed on 12 July 2022)). Rapidly growing genome sequences contribute to surveilling this fast-spreading pathogen and distinguishing emerging lineages [8,9,10,11,12]. In particular, lineage classification is a critical tool for monitoring variants of concern (VOCs) or variants of interest (VOIs) with reduced susceptibility to neutralizing antibodies or having higher transmissibility [13]. Research indicated that distinct SARS-CoV-2 lineages could play a pivotal role in developing drugs and designing vaccines by altering pathogenesis in infected hosts or virus tropism [14,15]. Therefore, the rapid identification of SARS-CoV-2 lineages, associated with different medical conditions and symptoms, has assisted in the long-term surveillance of this pathogen and is of utmost importance for updating SARS-CoV-2 vaccines [16,17,18,19].

Viral classification, allowing precise and unambiguous communication between researchers in different fields, is a challenging problem [20,21]. At present, many scientists are working on effectively categorizing SARS-CoV-2. The World Health Organization (WHO) recommended the use of the Greek alphabet such as Alpha, Beta, Gamma, Delta, and Omicron to classify SARS-CoV-2 genomes [22]. An early work by Chinese researchers identified two major lineages, L and S, based on two highly linked single nucleotides [14]. In addition, other developed sequence typing tools, Nextstrain [23], GISAID [7], Phylogenetic Assignment of Named Global Outbreak Lineages (Pangolin) [11,24], COVID-19 Genotyping Tool [15], and Genome Detective Coronavirus Typing Tool [25], are critical for tracking emerging diversity and spread of certain lineages. There are 25 Nextstrain clades, 1725 Pango lineages, and 11 GISAID clades as of 20 April 2022. Nonetheless, the phylogeny-based classification methods, such as GISAID [7] and Pangolin [11], demand huge computation time and memory consumption [18]. Moreover, those methods have a great demand for genetic distance thresholds when determining the maximal genetic differentiation among closely related viruses [18,26]. As for the single nucleotide polymorphism (SNP)-based classification methods, including Chinese lineage [14] and Nextstrain [23], are not enough to fully address the complex genetic diversity of SARS-CoV-2, for those two methods depend on mutations with significant geographic distribution and frequency or marker mutations [27]. Since the genetic diversity of SARS-CoV-2 challenges the current classification methods of SARS-CoV-2 variants [6], a more inexpensive, rapid, effective, and robust classification method is needed to identify the lineage of the virus, making it possible to quantitatively partition and describe the diversity of SARS-CoV-2 lineages [8,12,28,29,30]. Given that an impressive amount of sequencing data is being generated, we intend to adopt supervised learning-based approaches, which attempt to learn directly from the data, to classify SARS-CoV-2 genome sequences.

**Figure 1:**
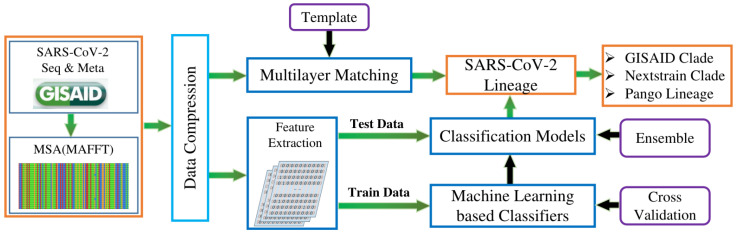
Processing pipeline of the SARS-CoV-2 genome sequence typing system. The system mainly includes data acquisition and preprocessing, multiple sequence alignment, data compression and feature extraction, supervised model training, and model testing. The ensemble model can achieve three different genome sequence typing predictions (GISAID, Nextstrain, and Pangolin). Templates can be obtained from the training set, and the Nextstrain or GISAID clade of the testing SARS-CoV-2 genome sequence can be obtained directly through the matching algorithm.

As shown in Figure 1, the proposed system in this study focuses on the rapid classification of SARS-CoV-2 genome sequences through supervised learning methods. Different from the previous work, the focus of this study is not to discover new evolutionary branches, but to provide a model with improved efficiency and accuracy based on existing Nextstrain, GISAID, and Pangolin classification standards. In summary, the main contributions of this study are listed as follows: (1) Supervised learning-based identification models are constructed for the three typing strategies of Nextstrain, GISAID, and Pangolin, respectively, achieving rapid and accurate SARS-CoV-2 genome sequence typing. (2) A multilayer template matching algorithm is proposed for SARS-CoV-2 genome sequence typing, achieving ideal results for the Nextstrain and GISAID clades. (3) Based on the template matching algorithm, this study has proposed a matching score-based method to quantify the difference between clades. (4) The lightweight data structure proposed in this study reduces the computational resource requirements of the model. (5) Finally, the ensemble model can achieve higher accuracy by fusing the prediction results of different methods. Extensive tests on a large amount of SARS-CoV-2 genome sequences show that the classification model constructed in this study has high accuracy and robustness. Furthermore, by introducing sub-models, this study can efficiently construct an extended model that identifies newly emerging clades.

## 2. Materials and Methods

### 2.1. Data Collection and Preprocessing

**Figure 2:**
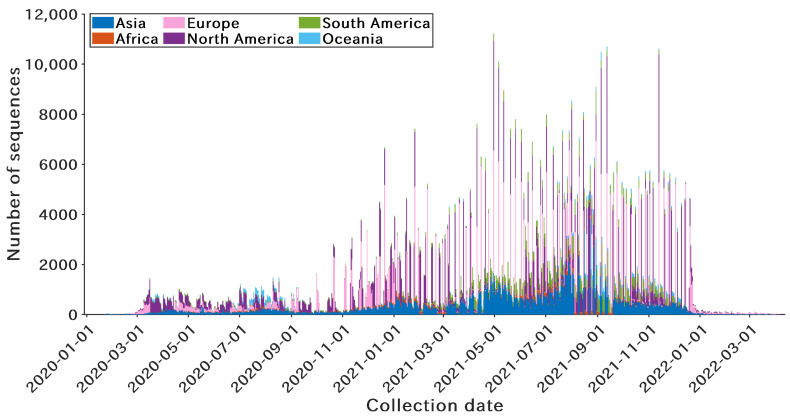
Temporal and spatial distribution of the extracted SARS-CoV-2 genome sequences. A total of >1 million SARS-CoV-2 genome sequences are downloaded from the GISAID database.

As of 20 April 2022, 1,088,952 complete SARS-CoV-2 genome sequences with high coverage [31] were extracted from the GISAID database (https://www.gisaid.org/ (accessed on 12 July 2022)). Notably, to improve the persuasiveness of the results, these sequences were extracted by the collection dates and regions (Figure 2). Given the different classification densities of the three typing tools (GISAID, Nextstrain, and Pangolin), sequences of each clade or lineage were uniformly sampled according to the collection date. Overall, the amounts of downsampled sequences are 91,772, 203,740, and 279,899 for GISAID, Nextstrain, and Pangolin, respectively.

The above nucleotide sequences were aligned with the reference sequence (Wuhan-Hu-1, NCBI accession NC_045512) using the option of “addfragments” in MAFFT version 7.490. Each sample was composed of the aligned nucleotide sequence and its designated label. For the GISAID and Pangolin, the designated clades or lineages were contained in the metadata of the corresponding genome sequences. For the Nextstrain part, labels of sequences were obtained from the Nextclade system (https://clades.nextstrain.org/ (accessed on 12 July 2022)). After this step, samples with classification labels were obtained. These samples were then divided into training sets (25%) and testing sets (75%).

### 2.2. Data Compression and Feature Extraction

As only part of the SARS-CoV-2 genomes have mutations [31], to reduce the computational cost, invariant sites of the aligned genome sequences were discarded [24] to obtain compressed sequences. We referred to the nucleotide site screening protocol provided by Nextclade (https://clades.nextstrain.org/ (accessed on 12 July 2022)) and PangoLEARN (https://github.com/cov-lineages/pangoLEARN/ (accessed on 12 July 2022)) and selected feature extraction strategies suitable for different typing methods through subsequent model training and cross-validation. Specifically, Nextclade defined each clade by the combination of signature mutations, providing a total of 83 mutations for 25 clades. On the other hand, PangoLEARN removed nucleotide sites without any SNPs and a total of 4544 sites were preserved.

**Figure 3:**
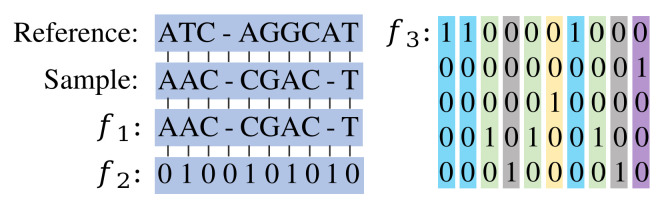
Examples of the three data structures applied in this study. $f_{1}$ is basically the same as the sample, and the unknown nucleotides in the sample are replaced with nucleotides at the corresponding locations in the reference sequence. $f_{2}$ is obtained by aligning the sample sequence with the reference sequence, thereby highlighting the mutation sites. $f_{3}$ has the largest amount of data, and each site is represented by a 5×1 vector.

Given the number of reserved nucleotide sites, we have tested three data structures (Figure 3):

(1) $f_{1}$ is with the size of n×1, where *n* is the number of reserved sites (n∈[83,4544]). $f_{1}$ contains five different values (A, T, G, C, and -), and those sites with unknown nucleotides due to sequencing errors were replaced with the nucleotides of the reference sequence [24].

(2) $f_{2}$ is with the same size as $f_{1}$. Element 1 indicates that the nucleotide type of this site is different from the reference sequence.

(3) $f_{3}$ is with the size of n×5. Each sequence was represented as a vector of one-hot encoded nucleotides [24].

It is noted that $f_{1}$ is only applied to the template matching method, while $f_{2}$ and $f_{3}$ are applied to machine learning-based methods. Details will be explained in the subsequent algorithm description section.

### 2.3. Template Matching Method

The template matching method was proposed for GISAID and Nextstrain clades. These two typing strategies have fewer branches (11 and 25, respectively) than Pangolin, avoiding the computational explosion of the matching algorithm. Furthermore, to balance the calculation efficiency and matching accuracy, a hierarchical matching algorithm is applied. Specifically, the template matching method is based on data structure $f_{1}$, and the exact matching score is computed by Hamming Distance [32]:

(1) $$d_{H}\left(A,B\right)=\sum_{i=1}^{N}\left(A\left(i\right)⊕B\left(i\right)\right),$$

where *N* is the number of selected sites, and ⊕ stands for the exclusive OR (XOR) operation. Based on (1), the exact matching score between the query sequence and one of the template sequences is defined as:

(2) $$S\left(Q,T_{j}\right)=1-\frac{d_{H}\left(Q,T_{j}\right)}{N},$$

where *Q* is the compressed query sequence, $T_{j}$ denotes the *j*th template sequence, and *N* is the number of reserved sites.

Templates can be obtained from the training set, and this work selected the sequence with the highest coverage as the template for the corresponding clade. The proposed multilayer matching algorithm is described in Algorithm 1, where $C_{l}^{*}$ and $C_{h}^{*}$ are the output clades at the low and high resolution of matching, respectively. For most query sequences, the exact matching (Step 2) at the high resolution is only performed twice, ensuring the accuracy and efficiency of the proposed algorithm.
**Algorithm 1** Two-level resolution template matching algorithm**  Require:** 
The compressed query sequence $Q_{l}$ and $Q_{h}$. Two groups of templates $\left\{T_{l}\right\}$ and $\left\{T_{h}\right\}$ with length $N_{l}$ and $N_{h}$. The number of templates for each group is denoted as $N_{t}$.  1:Initial matching scores: $S_{l}^{max}\leftarrow 0,S_{h}^{max}\leftarrow 0$.  2:Step 1: Exact matching between $Q_{l}$ and $\left\{T_{l}\right\}$:  3:**for**i=1 to $N_{t}$ **do**  4:     $S_{l}^{i}=1-\frac{d_{H}\left(Q_{l},T_{l}^{i}\right)}{N_{l}}$.  5:     **if** $S_{l}^{i}>S_{l}^{max}$ **then**  6:         $S_{l}^{max}\leftarrow S_{l}^{i},\;C_{l}^{max}\leftarrow C_{i}$, where $C_{i}$ is the *i*th clade.  7:     **end if**  8:**end for**  9:Sort $\left\{S_{l}^{i}\right\}$ in descending order: $\left\{S_{l}^{i^{\prime }}\right\}$. The superscripts $1^{\prime }$ and $2^{\prime }$ are the clade index numbers corresponding to the highest and next highest scores, respectively.10:**if**$S_{l}^{1^{{}^{\prime }}}>S_{l}^{2^{{}^{\prime }}}$**then**11:     $C_{l}^{*}\leftarrow C_{l}^{max}$.12:     **return** $C_{l}^{*}$.13:**else**14:     The clade index numbers with the same and the highest score form the set $I_{h}$, and the size of $I_{h}$ is $N_{t}^{h}$, where $N_{t}^{h}\geq 2$.15:     Continue with Step 2.16:**end if**17:Step 2: Exact matching between $Q_{h}$ and $\left\{T_{h}\right\}$:18:**for***i* in $I_{h}$ **do**19:     $S_{h}^{i}=1-\frac{d_{H}\left(Q_{h},T_{h}^{i}\right)}{N_{h}}$.20:     **if** $S_{h}^{i}>S_{h}^{max}$ **then**21:         $S_{h}^{max}\leftarrow S_{h}^{i},\;C_{h}^{max}\leftarrow C_{i}$, where $C_{i}$ is the *i*th clade.22:     **end if**23:**end for**24:$C_{h}^{*}\leftarrow C_{h}^{max}$.25:**return**$C_{h}^{*}$.

### 2.4. Difference Matrix between Clades

Based on the template matching method, this study proposed a difference matrix D to characterize the distance between clades. D is a diagonal matrix, and its element $D_{ij}$ is computed by (3), where *i* and *j* refer to two different clades, and $T_{i}$ and $T_{j}$ are the corresponding templates. $M_{1}$ and $M_{2}$ are the numbers of sequences that can be correctly classified with the feature length $N_{f}$.

(3) $$D_{ij}=\frac{1}{M_{1}+M_{2}}\left(\sum_{k=1}^{M_{1}}\frac{d_{H}\left(Q_{j}^{k},T_{i}\right)}{N_{f}}+\sum_{l=1}^{M_{2}}\frac{d_{H}\left(Q_{i}^{l},T_{j}\right)}{N_{f}}\right)$$

**Figure 4:**
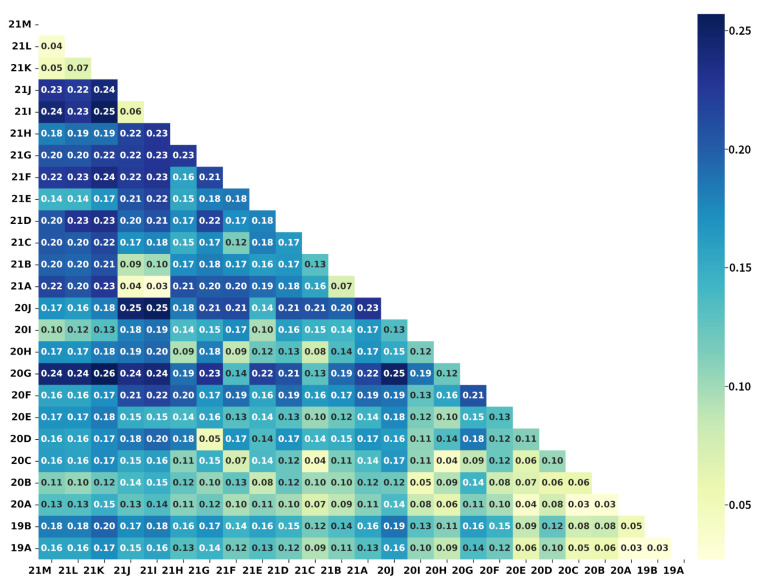
The difference matrix D of the Nextstrain clades. Each element in the matrix represents the difference between the corresponding two Nextstrain clades, and the dark color represents a larger difference.

The difference matrix D of the 25 Nextstrain clades is shown in Figure 4. A larger $D_{ij}$ implies a larger difference between clades *i* and *j*. D can not only be used to quantify differences between clades but also play a role in the evaluation of models.

### 2.5. Ensemble Learning-Based Classifier

Seven supervised classifiers were applied in this work, including Logistic Regression (LR), Decision Tree (DT), Random Forest (RF), Support Vector Machine (SVM), Multilayer Perceptron (MLP), Adaboost, and Catboost. Our goal is to screen out the optimal classification models and evaluate the performance of those classifiers on the two data structures $f_{2}$ and $f_{3}$, respectively. In addition, this study explored the ensemble of multiple models, such as the weighted fusion of multi-model predictions.

- LR is one of the most commonly used analytical methods in epidemiology and medicine [33]. As an extension of linear regression, LR is quite efficient with time and memory requirements, processing larger data with smaller resources. Using the one-vs.-rest (OvR) scheme, LR is applied for multiclass tasks. However, LR is prone to underfitting, resulting in low accuracy, especially in multi-classification tasks with unbalanced samples.
- DT adopts a tree structure for classification model training [34]. Starting from the root node, each branch divides the training data into disjoint subsets. The decision tree can be visualized and easily understood and interpreted. On the other hand, DT is prone to overfitting and is sensitive to data bias.
- RF is ensembled by multiple decision trees [24,35]. Each tree is built using a sub-set of the training sets. All decision trees vote on the classification, and the category with the most votes is the classification result of the RF. The RF prediction model can be trained fast and is easy to operate in parallel. In addition, the RF can output the feature importance computed as the total reduction of the criterion brought by that feature [36]. Previous studies have revealed that RF shows better performance than LR and DT in terms of SARS-CoV-2 clade classification [24,28].
- SVM solves the classification problem by finding the best hyperplane, which correctly divides the training sets and maximizes the geometric interval between the support vectors [37]. The hyperplane is presented as:
  (4) w·x+b=0,
  where *x* is the feature vector, and *w* and *b* represent the normal and intercept vectors of the hyperplane, respectively. By introducing kernel functions, SVM can solve nonlinear classification problems.
- MLP is an artificial neural network consisting of fully connected layers with at least one hidden layer [38]. Taking the case with one hidden layer as an example, the mathematical model of the MLP can be expressed as:
  (5) $$f\left(x\right)=w_{2}\cdot \phi \left(w_{1}\cdot x+b_{1}\right)+b_{2},$$
  where *x* is the input vector, $w_{1}$ and $w_{2}$ are the weights of input and hidden layers, $b_{1}$ and $b_{2}$ represent the bias vectors. ϕ is the activation function, such as the rectified linear unit function (ReLU) or the hyperbolic tan function (tanh).
- Adaboost is applied as a strong classifier constituted with multiple weak classifiers [39]. A base classifier is first trained from the initial training set, and the weights of training samples are then adjusted based on the training loss. As a result, the misclassified samples obtain more attention in subsequent training iterations. After *T* iterations of training, these *T* weak classifiers ($h_{t}\left(x\right)$) are weighted to form a strong classifier (H(x)), and a weak classifier with a smaller classification error has a larger weight $\alpha_{t}$:
  (6) $$H\left(x\right)=sign\left(\sum_{t=1}^{T}\alpha_{t}h_{t}\left(x\right)\right).$$
- Catboost is an algorithm for gradient boosting on decision trees [40,41]. Since the default parameters of Catboost provide great training results, it can reduce the time of parameter tuning. Catboost requires less hyperparameter tuning, reducing the possibility of overfitting and making the model more general. Additionally, Catboost supports model training on GPUs, improving training efficiency on large datasets. However, for the processing of categorical features, Catboost still consumes a lot of memory and time.

After training and testing the above models, on the one hand, this study analyzed the advantages and disadvantages of different models in the application of SARS-CoV-2 genome sequence typing and selected the optimal model; on the other hand, this study explored the combination of different models to obtain better prediction results than a single model. To facilitate the combination of the above methods, all classifiers were trained with enabled probability estimates. Furthermore, the proposed ensemble learning system applied weighted voting as the combination strategy [42]. Taking the Nextstrain clade typing task as an example, $p_{j}^{i}$ represents the probability that the *i*th classifier predicts the *j*th clade. The ensembled probability of the *j*th clade is computed as:

(7) $$P_{j}=\sum_{i=1}^{N}\omega_{i}\cdot p_{j}^{i},$$

where *N* is the number of ensembled models, and $\omega_{i}$ is the weight of the *i*th classifier, satisfying that $\sum \omega_{i}=1$. The ensembled sequence typing prediction result $C^{*}$ is determined with the maximum probability:

(8) $$C^{*}=\arg\;\underset{j}{\max}\left\{P_{j}\right\}.$$

### 2.6. Evaluation Metrics

To facilitate the internal verification and external testing of the model, this study mainly applies statistics including precision, recall, F-score, training and testing efficiency to quantify the model. In addition, for GISAID and Nextstrain, this study also applies the average difference $\bar{D}$ to test the classifier:

(9) $$\bar{D}=\frac{1}{N_{F}}\sum_{k=1}^{N_{F}}D_{ij}^{k},$$

where $N_{F}$ represents the number of samples with incorrect predictions.

## 3. Results

All experiments in this study were conducted on a portable laptop with an Intel Core i7 CPU (32G memory) at 2.30 GHz and an Nvidia RTX3070 GPU (8 G).

### 3.1. Feature Extraction

This study applied the sites provided by Nextstrain and Pangolin as the basis for feature extraction. As of 20 April 2022, Nextstrain provides 83 ($F_{min}$) nucleotide sites for the classification of 25 SARS-CoV-2 clades. On the other hand, Pangolin provides 4544 ($F_{max}$) nucleotide sites for the classification of over one thousand lineages. To meet the requirements of different typing strategies for accuracy, efficiency, and calculation memory usage, this study filtered the features and obtained different levels of feature scales in the range of [$F_{min}$, $F_{max}$].

**Figure 5:**
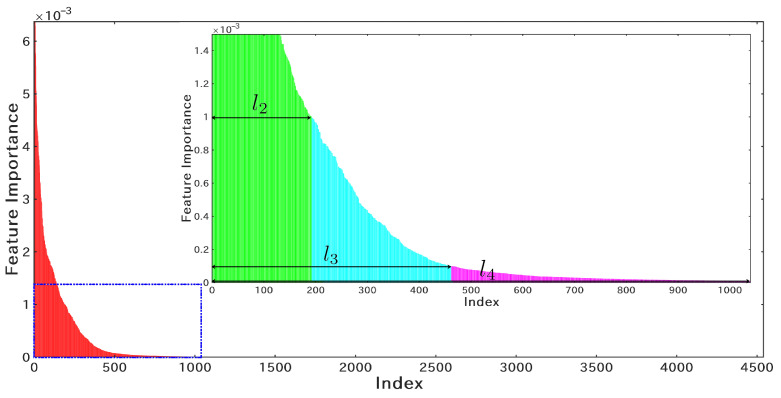
The feature importance distribution of the RF classifier obtained from the Nextstrain training dataset. The index is the result of sorting the sites in descending order of importance. $l_{2}$, $l_{3}$, and $l_{4}$ are obtained by setting the threshold of the feature importance to $10^{-3}$, $10^{-4}$, and $10^{-5}$.

Given the successful application of the RF classifier in Pangolin [24], the RF classifier was trained to classify the 25 clades of Nextstrain, and the initial feature scale was set to $l_{5}=F_{max}$. Then, the feature importance distribution was obtained as shown in Figure 5. Since the RF classifier adopts the one-hot method ($f_{3}$ in Section 2.2), each site corresponds to a 5×1 vector. Therefore, for the site weight calculation, the maximum value of the five features was used as the importance weight of the site. Taking $10^{-3}$, $10^{-4}$, and $10^{-5}$ as the thresholds, $l_{2}$, $l_{3}$, and $l_{4}$ were obtained, respectively. The corresponding numbers of effective sites are 192, 464, and 1048, respectively. Obviously, $l_{1}$ corresponds to the 83 effective sites provided by Nextstrain, and $l_{5}$ corresponds to the 4544 sites offered by Pangolin. It should be pointed out that GISAID has the least number of clades. To simplify the model training, this study applied the same feature settings as Nextstrain for the construction of the GISAID clade typing model.

### 3.2. Nextstrain Clade Typing Results

The datasets used for the Nextstrain typing experiment consist of two groups: the first group ($S_{1}^{N}$) includes 50,935 sequences for model training; the second group ($S_{2}^{N}$) includes 152,805 sequences for model testing.

#### 3.2.1. Training of the Nextstrain Clade Typing Models

**Figure 6:**
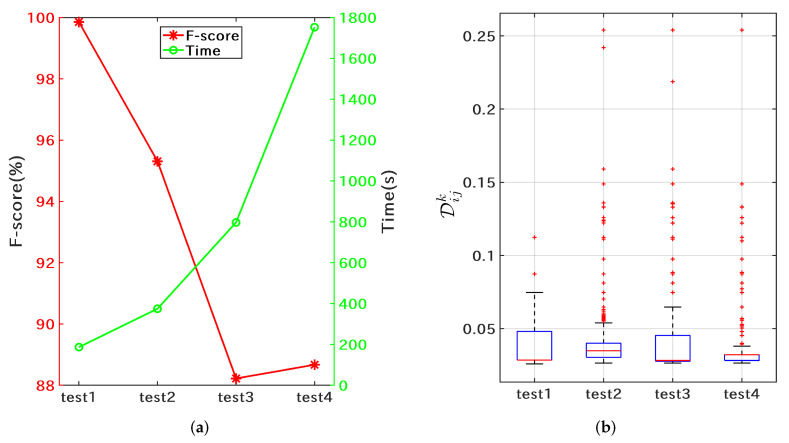
Template matching performance on the Nextstrain training set $S_{1}^{N}$. (**a**) the average F-scores (red) and the training time (green) for each of the tests; (**b**) difference statistics for misclassified samples.

Firstly, parameter optimization was performed for template matching using dataset $S_{1}^{N}$. As shown in Algorithm 1, hyperparameters of the proposed multilayer matching algorithm are mainly $N_{l}$ and $N_{h}$. $N_{h}$ was set to 4544 ($l_{5}$), and $N_{l}$ was set to four levels ($l_{1}$, $l_{2}$, $l_{3}$, $l_{4}$). As shown in Figure 6a, test1 ($N_{l}$ is set to $l_{1}=83$) obtains the highest matching accuracy and efficiency. As the number of features for coarse matching increases, the time-consuming increases; however, the accuracy decreases. At the same time, the number of misclassified samples with large differences (>1) also increases accordingly (Figure 6b). The template matching algorithm assigns all features the same score weight. Despite the shortcomings of this design, the proposed method has simple parameter settings and high matching efficiency, and subsequent experiments show that this method can match the performance of machine learning methods in sequence typing. In addition to the optimized parameter $N_{l}$ for the template matching algorithm, the difference matrix of the Nextstrain clades as shown in Figure 4 was also obtained.

Different from the template matching algorithm, the machine learning method can obtain the weights of features, so as to play the role of automatic screening of features in the training process. In light of RF’s excellent performance in SARS-CoV-2 clade classification [24], this part applied the training dataset $S_{1}^{N}$ to test the performance of the RF classifier with different numbers of features ($l_{1}$, $l_{2}$, $l_{3}$, $l_{4}$, and $l_{5}$) and data structures ($f_{2}$ and $f_{3}$).

**Figure 7:**
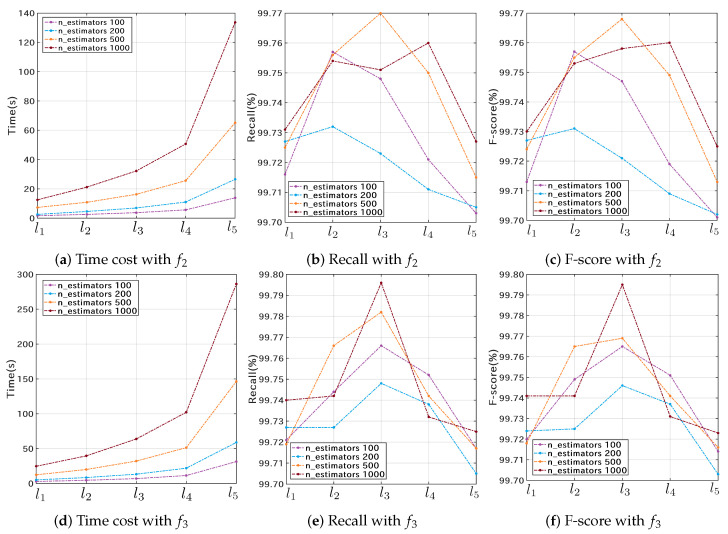
Cross-validation results of the RF classifier on the Nextstrain training dataset $S_{1}^{N}$. The first and second rows represent the validation results using the $f_{2}$ and $f_{3}$ data structures, respectively.

The 3-fold cross-validation results of the RF classifier are shown in Figure 7. The four curves in each sub-figure correspond to 100, 200, 500, and 1000 estimators, respectively. The recall and F-score curves show similar trends across different experimental groups. As the number of features increases, the training and validation time increases. In addition, the cross-validation based on data structure $f_{3}$ is much more time-consuming than that on $f_{2}$. However, the recall and F-score of $f_{2}$ are only marginally inferior to those of $f_{3}$, showing the superiority of the lightweight data structure $f_{2}$ for typing SARS-CoV-2 genome sequences. Overall, the group with feature size $l_{3}$, data structure $f_{3}$, and estimator number 1000 obtained the best classification accuracy (Figure 7e,f).

**Figure 8:**
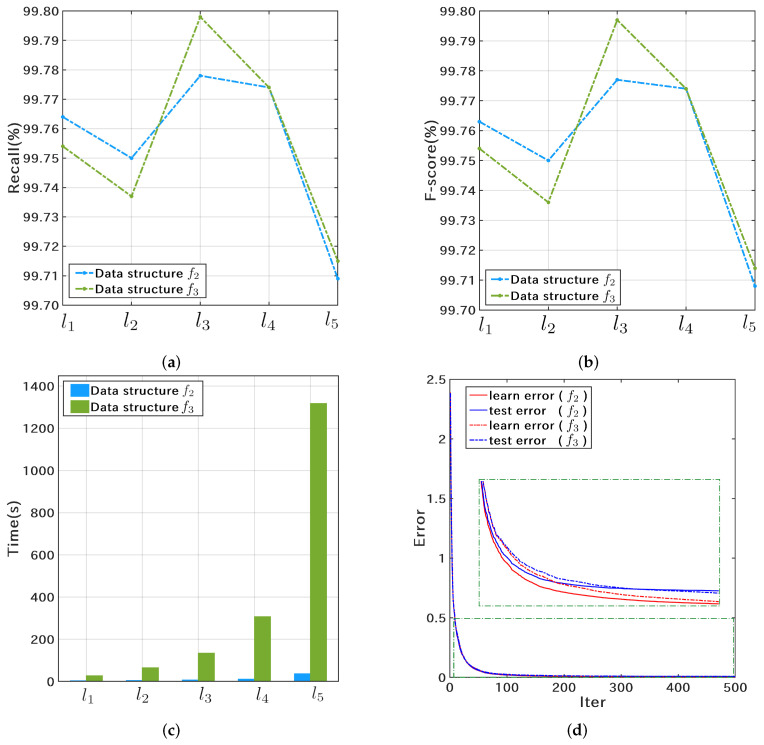
Three-fold cross-validation results of the Catboost classifier on the Nextstrain training dataset $S_{1}^{N}$. (**a**,**b**) show the recall and F-score curves; (**c**) shows the average time cost per cross-validation; (**d**) shows the learning and testing error curves.

Since Catboost has the advantages of rapid parameter tuning, high accuracy, low risk of overfitting, and is suitable for GPU-accelerated training [40,41], this study further analyzed the cross-validation results of the Catboost classifier. The results of the 3-fold cross-validation of the Catboost classifier on dataset $S_{1}^{N}$ are shown in Figure 8. The recall and F-score curves shown in Figure 8a,b indicate that the Catboost classifier is slightly better than the RF classifier. In addition, the best classification result is obtained at the number of features $l_{3}$. As for time cost, the advantage of $f_{2}$ is more prominent (Figure 8c). Figure 8d shows the curves of learning error and testing error in one of the cross-validations, and the horizontal axis represents the number of iterations.

The above two sets of experimental results show that the SARS-CoV-2 genome sequence typing based on both data structures $f_{2}$ and $f_{3}$ can achieve ideal results. The former ($f_{2}$) has a prominent advantage in efficiency, while the latter ($f_{3}$) has a slight advantage in accuracy. In addition, choosing the number of features as $l_{3}$ achieved ideal results in both accuracy and efficiency. The other five classifiers were trained by 3-fold cross-validation with the feature size of $l_{3}$. The cross-validation results of the seven models based on the training dataset $S_{1}^{N}$ are shown in Table 1, arranged in descending order of the F-score. $\bar{D}$ stands for the average difference computed by (9). Catboost, RF, and Adaboost obtained the top three classification accuracy. DT obtained the highest efficiency but the worst accuracy. In addition, the $\bar{D}$ of the seven models are all less than 0.08, and over half of them are less than 0.06, indicating that the misclassified samples mainly exist between clades with small differences.

#### 3.2.2. Testing of the Nextstrain Clade Typing Models

The dataset $S_{2}^{N}$ with 152,805 sequences was tested for external validation. In addition to the seven learning-based classifiers, this part tested the proposed template matching method (TM) and the ensemble model (Ensemble). Based on the cross-validation results in Section 3.2.1, the weights of classifiers in the ensemble model were set as: $\omega_{1}=0.25$ (Catboost), $\omega_{2}=0.25$ (RF), $\omega_{3}=0.2$ (Adaboost), $\omega_{4}=0.15$ (LR), $\omega_{5}=0.1$ (SVM), $\omega_{6}=0.05$ (MLP). DT obtained the worst classification measures (Table 1) and was not used for the ensemble method.

**Figure 9:**
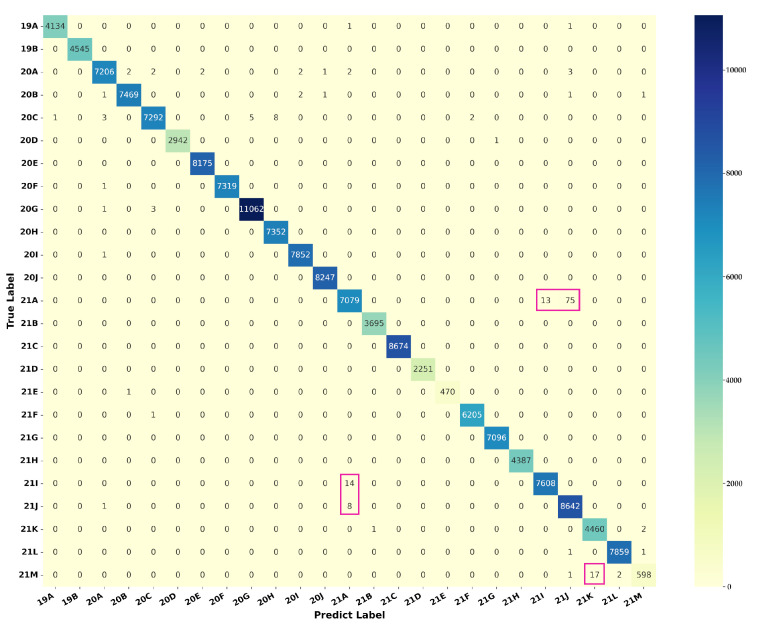
Confusion matrix produced by the ensemble model on the Nextstrain testing dataset $S_{2}^{N}$ using data structure $f_{3}$. The dark color represents a larger number of samples. Pink boxes mark easily misclassified samples distributed between clades with small differences.

Table 2 shows the results of the nine classification methods on dataset $S_{2}^{N}$, and the average testing time required for each aligned sequence is also represented in the table. Firstly, considering the precision, recall, and F-score measures, the Catboost classifier achieved the best performance among seven machine learning-based methods for both $f_{2}$ and $f_{3}$. RF, Adaboost, and LR also achieved ideal classification results for both $f_{2}$ and $f_{3}$. Secondly, methods using data structure $f_{2}$ were less time-consuming. RF, LR, and DT obtained higher accuracy and efficiency on data structure $f_{2}$. Thirdly, TM achieved better classification measures (precision, recall, and F-score) than any machine learning-based method. Finally, the ensemble model achieved the highest accuracy among all methods. The confusion matrix produced by the ensemble model based on $f_{3}$ is shown in Figure 9. Although the $f_{2}$-based ensemble method is slightly inferior to the $f_{3}$-based one on the three classification measures, the former is significantly more computationally efficient than the latter. In addition, except for DT, all methods, including TM and the ensemble model, obtained $\bar{D}<0.055$, indicating that the misclassified samples are mainly distributed among clades with small differences, like the Delta (21A, 21I, and 21J) and the Omicron (21M, 21K, and 21L) clades (as marked with pink boxes in Figure 9). To further compare the classification performance of different methods, the receiver operating characteristic (ROC) curves of different methods on dataset $S_{2}^{N}$ using data structure $f_{2}$ were plotted in Figure S1. The ensemble model obtained the best performance with the largest area under the curve (AUC).

### 3.3. GISAID Clade Typing Results

The experimental process of the GISAID clade typing is similar to Section 3.2. This part adopted the feature extraction method as described in Section 3.1 and conducted experiments on both $f_{2}$ and $f_{3}$ data structures. The datasets used for the GISAID typing experiment consist of two groups: the first group ($S_{1}^{G}$) includes 22,943 sequences for training; the second group ($S_{2}^{G}$) includes 68,829 sequences for testing.

#### 3.3.1. Training of the GISAID Clade Typing Models

**Figure 10:**
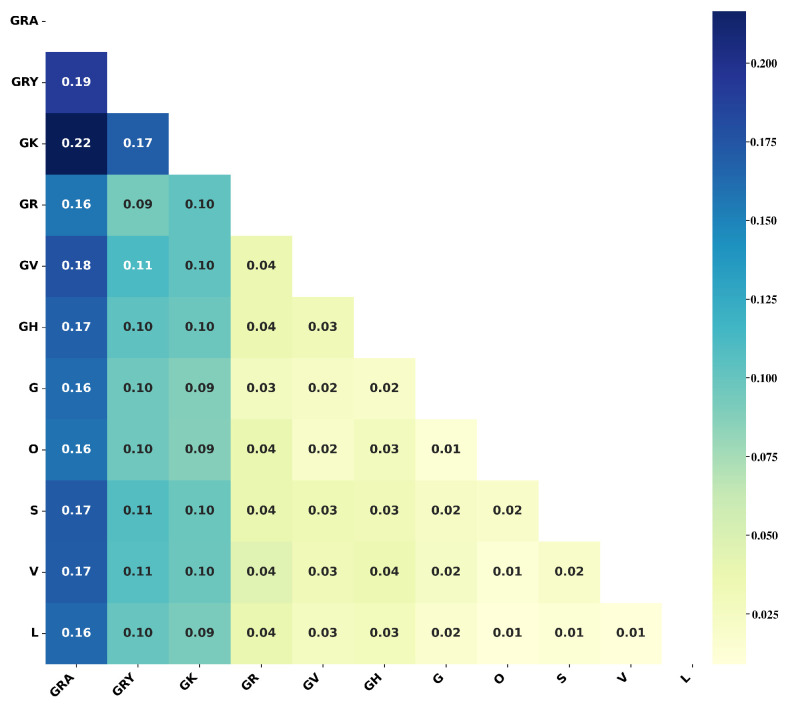
The difference matrix D of the GISAID clades. Each element in the matrix represents the difference between the corresponding two GISAID clades, and the dark color represents a larger difference.

There are 11 GISAID clades involved in this study (Figure 10). The difference matrix D was calculated based on the training dataset $S_{1}^{G}$ by Equation (3). Compared with Nextstrain’s difference matrix (Figure 4), GISAID’s D shows clear discrimination. Differences between the eight clades (L, V, S, O, G, GH, GV, and GR) in Figure 10 are quite small ($D_{ij}\leq 0.04$), while GK (Delta), GRY (Alpha), and GRA (Omicron) are quite different from other clades ($D_{ij}\geq 0.09$).

**Figure 11:**
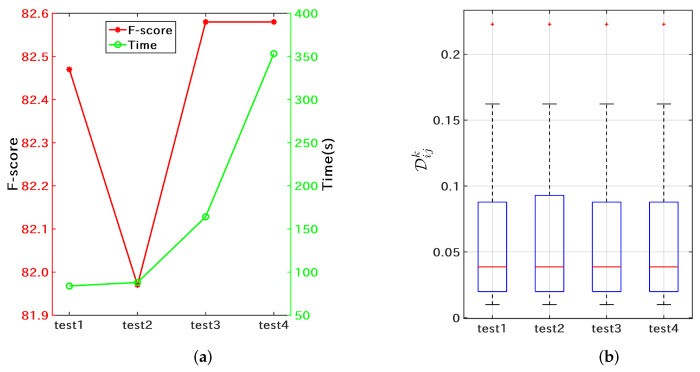
Template matching performance on the GISAID training set $S_{1}^{G}$. (**a**) the average F-score (red) and the training time (green) for each of the tests; (**b**) difference statistics for misclassified samples.

Firstly, the hyperparameters $N_{l}$ and $N_{h}$ of the proposed multilayer matching Algorithm (1) were set based on $S_{1}^{G}$. $N_{h}$ was set to 4544, and $N_{l}$ was set to four levels ($l_{1}$, $l_{2}$, $l_{3}$, and $l_{4}$). The corresponding four sets of training results are shown in Figure 11. Test3 and test4 obtained the same F-score, and the former was much more efficient. In addition, Figure 11b shows that there is no significant difference in the distribution of $D_{ij}$. Based on the above results, the parameter $N_{l}$ of the template matching method for GISAID clade typing was set to $l_{3}$.

**Figure 12:**
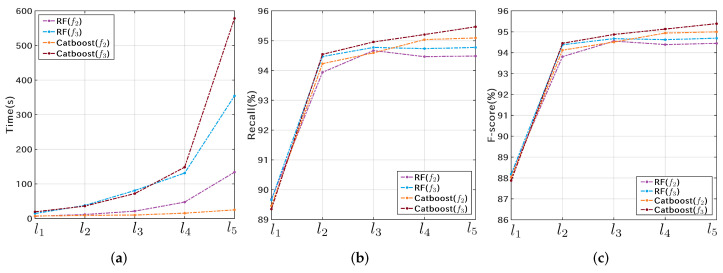
Cross-validation results of the RF and Catboost classifiers on the GISAID training dataset $S_{1}^{G}$. (**a**) the average time cost per cross-validation; (**b**) the recall rates of the RF and Catboost classifiers on data structures $f_{2}$ and $f_{3}$; (**c**) the F-scores of the RF and Catboost classifiers on data structures $f_{2}$ and $f_{3}$.

Secondly, the 3-fold cross-validation was applied to the machine learning-based methods. Based on the good performance of the RF and Catboost classifiers in the Nextstrain clade typing, we applied these two methods for feature scale selection. The cross-validation results based on five different feature scales and two data structures are shown in Figure 12. Overall, as the number of features increases, the training time increases, and the recall and F-score increase. Furthermore, the model with data structure $f_{3}$ slightly outperforms $f_{2}$ in terms of recall and F-score. To this end, the number of features used for the GISAID clade classification was set to $l_{5}$.

The cross-validation results of the seven models based on the training dataset $S_{1}^{G}$ are shown in Table 3. They are sorted in descending order by the F-score. All six other methods except MLP achieved higher recall rates and F-scores on data structure $f_{3}$. DT obtained the highest efficiency, but the second-to-last accuracy. SVM obtained the lowest accuracy on both $f_{2}$ and $f_{3}$ data structures. Furthermore, the accuracy of the GISAID clade typing is lower than that of Nextstrain, and the $\bar{D}$ of misclassified samples of the GISAID clade typing is larger.

#### 3.3.2. Testing of the GISAID Clade Typing Models

Further external validation was conducted to compare different typing models, using the dataset $S_{2}^{G}$ with 68,829 sequences. In addition to the seven supervised learning-based methods, TM and the ensemble model were also tested. Different from Nextstrain, the number of features used by the GISAID classification models is $l_{5}$. DT and SVM with the worst classification accuracy were removed from the ensemble model. Based on the cross-validation results shown in Table 3, the weights of the five classifiers in the ensemble model were set as: $\omega_{1}=0.25$ (Catboost), $\omega_{2}=0.25$ (MLP), $\omega_{3}=0.20$ (LR), $\omega_{4}=0.20$ (RF), $\omega_{5}=0.10$ (Adaboost).

**Figure 13:**
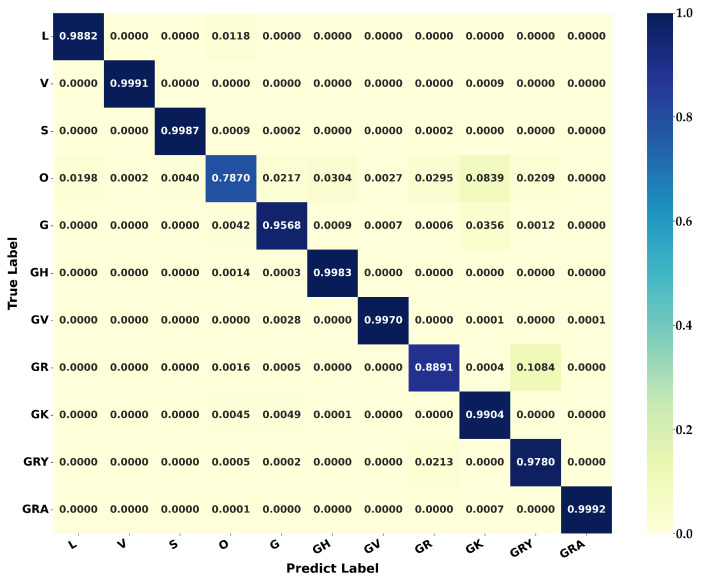
Confusion matrix produced by the ensemble model on the GISAID testing dataset $S_{2}^{G}$ using data structure $f_{2}$. The dark color represents a larger proportion.

Table 4 shows the results of nine classification methods on the testing dataset $S_{2}^{G}$, and the average testing time per sequence is also presented. Firstly, considering precision, recall, and F-score, the RF classifier achieved the best performance among those seven machine learning-based methods on both $f_{2}$ and $f_{3}$, followed by Catboost, LR, Adaboost, and MLP. TM is inferior to other models in the precision, recall, and F-score. However, its $\bar{D}$ is smaller, indicating that the misclassified samples of TM are mainly distributed between clades with small differences. Notably, the seven machine learning-based models have very little difference in accuracy between $f_{2}$ and $f_{3}$. Moreover, the ensemble model achieved the highest precision, recall, and F-score on $f_{2}$. In terms of computational efficiency, the prediction time per sample of the ensemble model on $f_{2}$ was only 31.7% of that on $f_{3}$, providing an accurate and efficient solution for the GISAID clade typing. The confusion matrix produced by the ensemble model on $f_{2}$ is shown in Figure 13. To facilitate comparison, elements in the matrix are expressed as proportions. Among them, the recall rates of clades O and GR are less than 90% and the recall rate of clade O is the lowest (78.7%). Figure S2 shows the ROC curves of different methods on dataset $S_{2}^{G}$ using data structure $f_{2}$. The ensemble model obtained the largest AUC.

### 3.4. Pango Lineage Typing Results

Unlike the Nextstrain and GISAID typing issues, the number of lineages defined by Pangolin is significantly increased [24], and TM is no longer suitable for Pango lineage typing. Due to a large number of lineages, the time-consuming and computational cost of model training increases significantly. Further considering the results in Section 3.2 and Section 3.3 and the performance of pangoLEARN [24], this study mainly applied RF and Catboost to conduct the Pango lineage typing research. In view of the validity of the model and the limitation of computing resources, we set the minimum number of samples of each lineage to 50, and a total of 710 lineages were obtained (lineages with less than 50 samples were discarded). In addition, no more than 2000 samples were screened for each lineage. Finally, a total of 279,899 sequences (69,565 training samples ($S_{1}^{P}$) and 210,334 testing samples ($S_{2}^{P}$)) were obtained.

#### 3.4.1. Training of the Pango Lineage Typing Models

**Figure 14:**
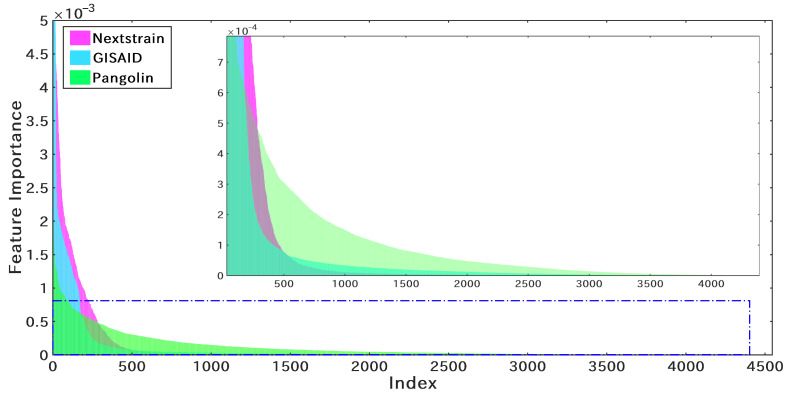
The feature importance distribution of three SARS-CoV-2 genome sequence typing strategies. The index is the result of sorting the sites in descending order of importance.

$S_{1}^{P}$ was applied to build the RF classifier, achieving the feature importance distribution (shown in Figure 14). Comparing it with the feature distributions of Nextstrain and GISAID, Pangolin (green) has the widest distribution of effective features, with only 53 of the 4544 sites weighting 0. Therefore, this study adopted the same setting as PangoLEARN [24] in the number of effective nucleotide sites ($l_{5}=4544$).

**Figure 15:**
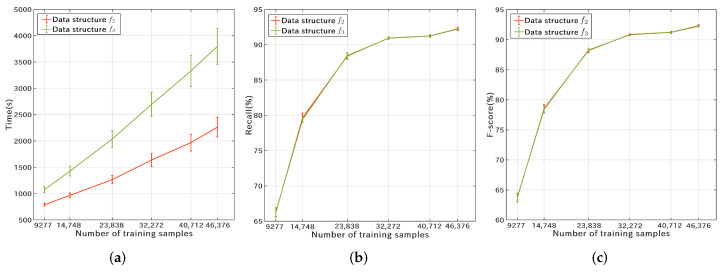
Cross-validation results of the RF classifier on the Pangolin training dataset $S_{1}^{P}$. (**a**) the time cost per cross-validation; (**b**) the recall rates of the RF classifier on data structures $f_{2}$ and $f_{3}$; (**c**) the F-scores of the RF classifier on data structures $f_{2}$ and $f_{3}$.

Due to a large number of Pango lineages, a larger amount of training samples require more computing resources and training time. To this end, we further downsampled $S_{1}^{P}$ to obtain six sets of training samples by setting the maximum number of samples ($N_{max}$) for each lineage. $N_{max}$ was set to 25, 50, 100, 200, 300, and 500, and the corresponding training dataset size ($N_{train}$) was 13,916, 22,123, 35,757, 48,408, 61,069, and 69,565. The 3-fold cross-validation results on $f_{2}$ and $f_{3}$ are shown in Figure 15. The horizontal axis in Figure 15 represents the number of samples involved in training in each cross-validation, which is 66.67% of $N_{train}$. Figure 15a shows that the training time is positively correlated with the number of samples, and the training on $f_{2}$ is more efficient. Figure 15b,c show that the F-scores and recall rates have very similar trends, and the classification performance on the two data structures differs very little. Considering both accuracy and efficiency, $f_{2}$ performs better than $f_{3}$ in Pango lineage typing. Further experiments showed that Catboost had very close validation results to RF.

#### 3.4.2. Testing of the Pango Lineage Typing Models

**Figure 16:**
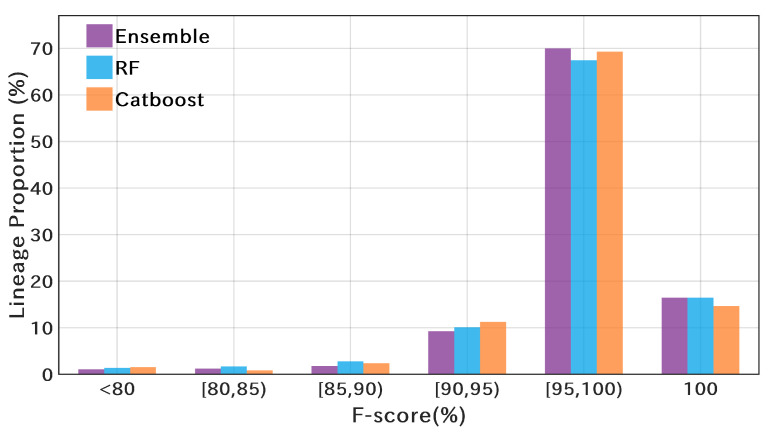
F-score distribution of different models on Pango lineage classification. The vertical axis represents the proportion of the Pango lineages in different F-score intervals.

We applied RF and Catboost to construct the Pango lineage classifiers and integrated the prediction results of the two models. To improve the accuracy of the classifiers, all samples in $S_{1}^{P}$ (69,565) were used for model training. The testing results on the dataset $S_{2}^{P}$ (210,334) are shown in Table 5. Furthermore, in the ensemble model, the weights of the prediction results of both models (RF and Catboost) were set to 0.5. It is worth noting that the RF classifier on data structure $f_{3}$ is the same as that applied by PangoLEARN [24] and can be used for comparison. As shown in Table 5, the proposed ensemble model on data structure $f_{2}$ achieved the highest classification precision, recall, and F-score. The ensemble model improved the classification accuracy on both data structures and achieved better performance on $f_{2}$ with less computation. Figure 16 shows the F-score distribution of the three models. The vertical axis represents the proportion of the Pango lineages in different F-score intervals. The ensemble model obtains the highest proportion of lineages with an F-score ≥95%. Furthermore, Figure S3 shows the ROC curves of the three methods on dataset $S_{2}^{P}$ using data structure $f_{2}$. The ensemble model outperforms RF and Catboost with a larger AUC.

### 3.5. Model Extension with Newly Emerging Clades

To deal with the classification of newly emerging clades, we tried to obtain an extended model by training a sub-model based on the existing model. Taking Nextstrain as an example, we obtained 561 sequences with high coverage of two new clades (22A (Omicron) and 22B (Omicron)) from the GISAID database. The collection dates of these sequences are from 25 April 2022 to 12 July 2022. According to Nextclade (https://clades.nextstrain.org/ (accessed on 12 July 2022)), 22A (Omicron) and 22B (Omicron) evolved from 21L (Omicron). For brevity, they are abbreviated as 22A, 22B, and 21L.

We applied the same method as the main model (Section 2.5) to construct a sub-model for the classification of the three clades (21L, 22A, and 22B). An extended model is composed of the main model and a sub-model. For sequences to be classified, we firstly applied the main model for classification. For the sample whose main model output was 21L, we continued to apply the sub-model for further classification. To this end, an extended classification model capable of handling newly emerging clades can be obtained with only a small amount of work. It should be pointed out that the construction of the sub-model needs to incorporate the nucleotide mutation sites of the two new clades 22A and 22B based on the original features. In this study, the training set (22A and 22B) was compared with the reference sequence, and the nucleotide sites with mutation rates exceeding a certain threshold were extracted. As a result, 35 additional feature points were obtained.

The resulting confusion matrix (Table 6) on the testing dataset shows that only four samples were mistakenly assigned to another clade by the sub-model, achieving a weighted accuracy of 99.519%. Furthermore, out of all 831 samples, only one sample with clade 21L was misassigned to 21M by the main model. All samples of clades 22A and 22B were correctly assigned to their father clade 21L by the main model. The training of the sub-model took only a few minutes and the extra testing time for each aligned sequence was less than 10 ms. Therefore, by introducing the sub-model, this study can rapidly construct an extended model that accurately identifies newly emerging clades.

## 4. Discussion

Facing the SARS-CoV-2 genome sequence typing problem, this study built classifiers for three typing strategies of GISAID, Nextstrain, and Pangolin. In addition to the machine learning-based methods, this study has proposed a method based on template matching for GISAID and Nextstrain. Based on the template matching algorithm, we obtained the difference matrix between viral clades and applied it as one of the classifier evaluation indicators. To achieve a fast and accurate classifier, two improvements have been made. First, two data structures based on one-hot coding and site mutation were used for nucleotide sequence transformation. Second, a weighted fusion strategy was applied to obtain an ensemble model. Overall, our study achieved the highest accuracy on Nextstrain clade typing (precision: 99.879%, recall: 99.879%, F-score: 99.879%), followed by the Pangolin (precision: 97.889%, recall: 97.732%, F-score: 97.766%) and the GISAID (precision: 96.433%, recall: 96.291%, F-score: 96.235%).

(1) Nextstrain: Our study has studied the classification of 25 Nextstrain clades, using seven machine learning-based methods and a template matching-based method. The ensemble model achieved the highest classification precision, recall, and F-score. The template matching algorithm achieved a classification performance comparable to any machine learning-based classifier. In addition, the difference matrix D obtained from the matching algorithm can intuitively represent the distance between different clades. Figure 4 and Figure 9 show that the misclassified samples are mainly distributed between clades with small differences. Furthermore, data structure $f_{2}$ has a better classification performance in SARS-CoV-2 genome sequence typing. Although the accuracy is slightly lower than that of $f_{3}$, the computational efficiency is improved by more than five times (as shown in Table 2).

(2) GISAID: Research on the classification of 11 GISAID clades has been carried out in this work. The ensemble model on data structure $f_{3}$ achieved the best results. Compared with the Nextstrain clade typing, TM performs worse in the GISAID clade classification, and the F-score is lower than 85%. Figure 10 shows that except for GRA, GRY, and GK, the GISAID clades are less diverse ($D_{ij}\leq 0.04$). Furthermore, a total of 13 (23.6%) elements in Figure 10 are less than 0.03, while those in Figure 4 equal zero. It indicates that the separability between GISAID clades is lower than that of Nextstrain clades. The ensemble model on $f_{3}$ obtained the highest typing accuracy with an ideal computational speed.

(3) Pangolin: A total of 710 Pango lineages are included in this study. The classification accuracy of RF and Catboost is very close, and the ensemble of the two methods can obtain higher precision, recall, and F-score. More interestingly, the performance of the ensemble model on $f_{2}$ is better than that on $f_{3}$, with higher accuracy and less computation time.

Compared with existing SARS-CoV-2 typing studies, our results have both improvements and limitations. The Genome Detective Coronavirus Typing Tool [25] can only identify the SARS-CoV-2 clades of several VOCs. In addition, this method is computationally inefficient, taking an average of 30 ms per genome. UShER [43] places sequences on a comprehensive tree and supplied sequences need to be uploaded to UShER’s servers where processing takes place. In addition, it takes an average of 18 ms to place one sample onto the reference tree using 16 threads and achieves an accuracy of 98.5% for samples with one parsimony-optimal placement. On the other hand, Nextclade [44] is an open-source project for viral genome alignment, mutation calling, clade assignment, quality checks, and phylogenetic placement. Although its web version can provide comprehensive and up-to-date sequence analysis results, its offline version performs clade assignment based on a small number of valid nucleotide sites, with low accuracy, and partial sequences cannot be effectively identified. Compared with Nextclade and UShER, this study does not construct the evolutionary tree but focuses on the typing of genomes. In addition, the methods proposed in this study (the template matching and the ensemble model) are computationally efficient (<20 ms for one sample) with higher accuracy (>99.85%). The disadvantage of our work is that we can only identify existing clades and cannot discover new SARS-CoV-2 clades. However, the proposed extended model can identify newly emerging clades by training sub-models with only a small amount of work.

As for the GISAID clade typing, its classification accuracy is relatively low. GISAID classification is based more on several marker variants than strictly phylogenetic relationships [18]. Moreover, clade O refers to other clades that do not meet the GISAID clade definition [45]. This can further explain that the typing model has the worst accuracy on clade O (recall: 77.249%, F-score: 86.625%). The PhenoGraph [46] classification identifies 303 SARS-CoV-2 clades and is consistent with, but more detailed and precise, than the known GISAID clades [18]. It provides an unsupervised clustering method for SARS-CoV-2 clades. In contrast, we provide supervised models for a different classification density. Although the weighted recall of the proposed model is about 96%, VOCs such as GK (Delta) and GRA (Omicron) can achieve an accuracy of over 99%.

The Pangolin classification tool [24] provides the basis for the research in this study. Different from PangoLEARN, this study tried a lightweight data structure $f_{2}$ with higher efficiency. The classification accuracy has been improved through model integration. The limitation of our method is that only 710 SARS-CoV-2 lineages are included in this study due to the constraints of computational resources. This problem can be solved by increasing the hardware configuration level and downloading more data. In addition, GNU-based Virus IDentification (GNUVID) is applied to assign sequence type profiles to all high-quality SARS-CoV-2 genomes [28]. The overall prediction statistics of GNUVID on high-quality genomes are precision (94.7%), recall (96.4%), and F-score (95.0%), which are lower than those of the classifier proposed in this study. In addition, this study adopts the lightweight data structure $f_{2}$ to improve the classification efficiency, and the average time per sequence is about 10 ms, which is much lower than the 31 ms of GNUVID [28].

## 5. Conclusions

This study presents a SARS-CoV-2 genome sequence classification system based on supervised learning methods. Overall, the system aims to achieve rapid and accurate SARS-CoV-2 genome sequence typing for the three typing strategies of Nextstrain, GISAID, and Pangolin, respectively. When we obtained SARS-CoV-2 genome sequences from COVID-19 patients, the system proposed in this study can be applied to efficiently and accurately type these sequences, which would help to carry out relevant epidemiological analysis and provide reliable typing and traceability basis for effectively blocking its spread. For Nextstrain and GISAID, this study has proposed a method based on template matching. Through the strategy of multi-layer matching, we improved the efficiency of the matching algorithm. The template matching method achieved satisfactory results in the Nextstrain clade typing. A template matching-based difference metric method is proposed to quantify the difference between two clades and serve as an evaluation factor for classifier performance. Furthermore, we have proposed an ensemble model that integrates a combination of machine learning methods (such as Random Forest and Catboost) with optimized weights. In addition to the one-hot coding method, this study has proposed a data structure based on nucleotide site mutation, which obtains good results in SARS-CoV-2 genome sequence typing. While obtaining ideal classification accuracy, the computational resources are greatly reduced. Finally, verified by a large number of testing datasets, the ensemble model proposed in this study helps to improve the accuracy of the classification system (Nextstrain: 99.879%, Pangolin: 97.732%, GISAID: 96.291%). This study provides a comprehensive and efficient method for SARS-CoV-2 genome sequence typing, which helps to monitor the diversity of SARS-CoV-2, thereby serving the global anti-epidemic. In addition, by introducing sub-models, this study can rapidly construct an extended model that accurately identifies newly emerging clades without retraining the main model constantly. Future work will focus on the discovery of new clades and the identification of recombination.

## Figures and Tables

**Table 1:** The cross-validation results of the seven classifiers on the Nextstrain training dataset $S_{1}^{N}$.

	Data Structure $f_{2}$					Data Structure $f_{3}$				
**Approach**	**Precision (%)**	**Recall (%)**	**F-Score (%)**	$\bar{D}$	**Time (s)**	**Precision (%)**	**Recall (%)**	**F-Score (%)**	$\bar{D}$	**Time (s)**
Catboost	99.776	99.778	99.777	0.048	6.6	99.796	99.798	99.797	0.047	134.9
RF	99.765	99.751	99.758	0.047	32.2	99.794	99.796	99.795	0.048	63.8
Adaboost	99.690	99.692	99.691	0.057	250.2	99.761	99.760	99.760	0.061	715.6
LR	99.714	99.716	99.715	0.051	17.3	99.740	99.726	99.733	0.048	79.8
SVM	99.711	99.713	99.712	0.048	9.6	99.727	99.729	99.728	0.047	63.5
MLP	99.641	99.645	99.643	0.067	34.9	99.674	99.676	99.675	0.066	167.2
DT	99.486	99.486	99.486	0.078	1.0	99.491	99.491	99.491	0.079	2.1

**Table 2:** Results of the nine classification methods on the Nextstrain testing dataset $S_{2}^{N}$.

	Data Structure $f_{2}$					Data Structure $f_{3}$				
**Approach**	**Precision (%)**	**Recall (%)**	**F-Score (%)**	$\bar{D}$	**Time (ms)**	**Precision (%)**	**Recall (%)**	**F-Score (%)**	$\bar{D}$	**Time (ms)**
Catboost	99.839	99.840	99.839	0.052	0.05	99.854	99.854	99.854	0.054	2.64
RF	99.823	99.824	99.823	0.047	0.19	99.817	99.816	99.816	0.048	0.39
Adaboost	99.810	99.809	99.810	0.051	0.84	99.831	99.831	99.830	0.054	2.18
LR	99.816	99.816	99.816	0.048	<0.01	99.809	99.809	99.808	0.051	<0.01
SVM	99.742	99.741	99.741	0.046	0.44	99.797	99.798	99.797	0.049	4.66
MLP	99.669	99.667	99.665	0.045	<0.01	99.792	99.786	99.789	0.044	0.01
DT	99.687	99.686	99.686	0.077	<0.01	99.664	99.664	99.664	0.078	<0.01
Ensemble	99.876	99.876	99.876	0.046	1.64	99.879	99.879	99.879	0.051	9.79
TM	Precision: 99.858%, Recall: 99.855%, F-score: 99.856%, $\bar{D}$: 0.049, Time: 4.76 ms									

**Table 3:** The cross-validation results of the seven classifiers based on the GISAID training dataset $S_{1}^{G}$.

	Data Structure $f_{2}$					Data Structure $f_{3}$				
**Approach**	**Precision (%)**	**Recall (%)**	**F-Score (%)**	$\bar{D}$	**Time (s)**	**Precision (%)**	**Recall (%)**	**F-Score (%)**	$\bar{D}$	**Time (s)**
Catboost	95.247	95.092	94.999	0.074	24.6	95.660	95.467	95.389	0.071	603.9
MLP	95.437	95.280	95.200	0.072	714.2	95.208	95.110	95.017	0.069	1487.1
LR	94.903	94.896	94.780	0.070	62.3	95.068	94.921	94.866	0.072	294.7
RF	94.547	94.465	94.364	0.071	133.8	94.853	94.735	94.621	0.070	707.7
Adaboost	94.022	94.012	93.978	0.072	1005.2	94.591	94.574	94.536	0.074	3581.5
DT	93.337	93.405	93.365	0.073	4.5	93.870	93.902	93.871	0.075	10.3
SVM	93.268	92.927	92.850	0.073	211.8	93.489	92.861	92.399	0.076	321.8

**Table 4:** Results of the nine classification methods on the GISAID testing dataset $S_{2}^{G}$.

	Data Structure $f_{2}$					Data Structure $f_{3}$				
**Approach**	**Precision (%)**	**Recall (%)**	**F-Score (%)**	$\bar{D}$	**Time (ms)**	**Precision (%)**	**Recall (%)**	**F-Score (%)**	$\bar{D}$	**Time (ms)**
RF	96.082	96.019	95.966	0.072	1.06	96.162	96.093	96.039	0.072	1.31
Catboost	96.064	95.928	95.860	0.073	0.57	96.130	95.983	95.920	0.073	2.17
LR	95.745	95.628	95.554	0.071	0.02	95.981	95.890	95.831	0.072	0.13
Adaboost	95.592	95.595	95.566	0.074	3.26	95.685	95.689	95.644	0.073	11.74
MLP	95.849	95.768	95.708	0.072	0.02	95.678	95.727	95.613	0.071	0.25
DT	95.029	95.046	95.031	0.073	0.01	95.461	95.476	95.455	0.074	0.02
SVM	95.112	94.910	94.846	0.074	27.54	93.916	93.286	92.889	0.078	143.63
Ensemble	96.433	96.291	96.235	0.074	5.00	96.357	96.140	96.066	0.074	15.75
TM	Precision: 88.366%, Recall: 85.203%, F-score: 82.540%, $\bar{D}$: 0.055, Time: 7.04 ms									

**Table 5:** Results of the Pango lineage classifiers on the testing dataset $S_{2}^{P}$.

	Data Structure $f_{2}$				Data Structure $f_{3}$			
**Approach**	**Precision (%)**	**Recall (%)**	**F-Score (%)**	**Time (ms)**	**Precision (%)**	**Recall (%)**	**F-Score (%)**	**Time (ms)**
RF	97.687	97.469	97.509	0.39	97.696	97.474	97.515	0.44
Catboost	97.667	97.519	97.554	0.66	97.588	97.425	97.464	1.85
Ensemble	97.889	97.732	97.766	1.69	97.868	97.715	97.746	3.01

**Table 6:** Confusion matrix resulting from the test of the sub-model, comprising 831 samples belonging to 3 clades (21L, 22A, and 22B).

		Predicted Label		
		**21L**	**22A**	**22B**
	**21L**	409	2	0
**True Label**	**22A**	0	240	0
	**22B**	1	1	178

## Data Availability

Codes and models are available at https://github.com/MiaoMiaorrk/SARS-CoV-2-Genome-Sequence-Typing (accessed on 30 July 2022) and SARS-CoV-2 whole-genome sequences were obtained from the Global Initiative on Sharing All Individual Data (GISAID) (https://www.gisaid.org/ (accessed on 12 July 2022)).

## Supplementary Materials

The following supporting information can be downloaded at: https://www.mdpi.com/article/10.3390/microorganisms10091785/s1, A list of SARS-CoV-2 genome sequence acknowledgments is provided in Data S1. Figure S1: ROC curves on the Nextstrain testing dataset, Figure S2: ROC curves on the GISAID testing dataset, Figure S3: ROC curves on the Pangolin testing dataset.
