# Supplementary material for: Towards Efficient and Accurate SARS-CoV-2 Genome Sequence Typing Based on Supervised Learning Approaches
Source: Microorganisms. 2022 Sep 4;10(9):1785. doi: 10.3390/microorganisms10091785 (PMC9505117; doi:10.3390/microorganisms10091785)
Supplement: Supplementary file 1 [file microorganisms-10-01785-s001.zip › microorganisms-1886349-supplementary-figures.pdf]

microorganisms

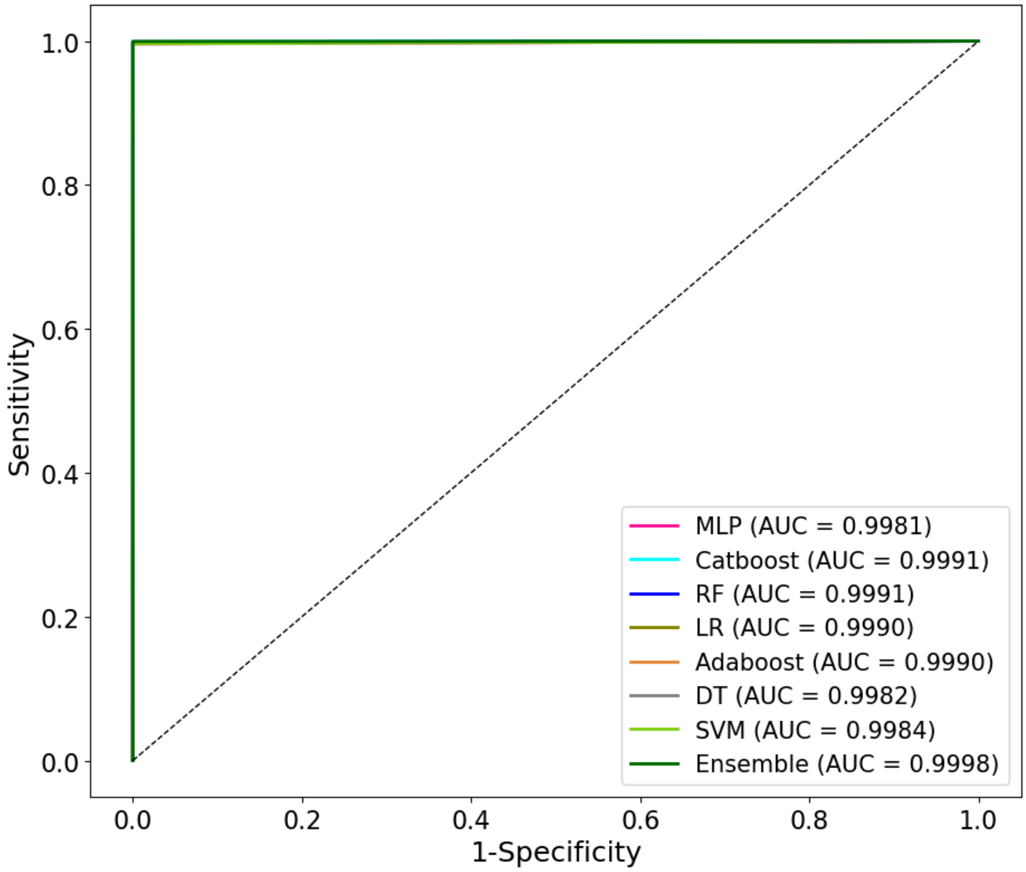

Figure S1. ROC curves of different classification methods on the Nextstrain testing dataset.

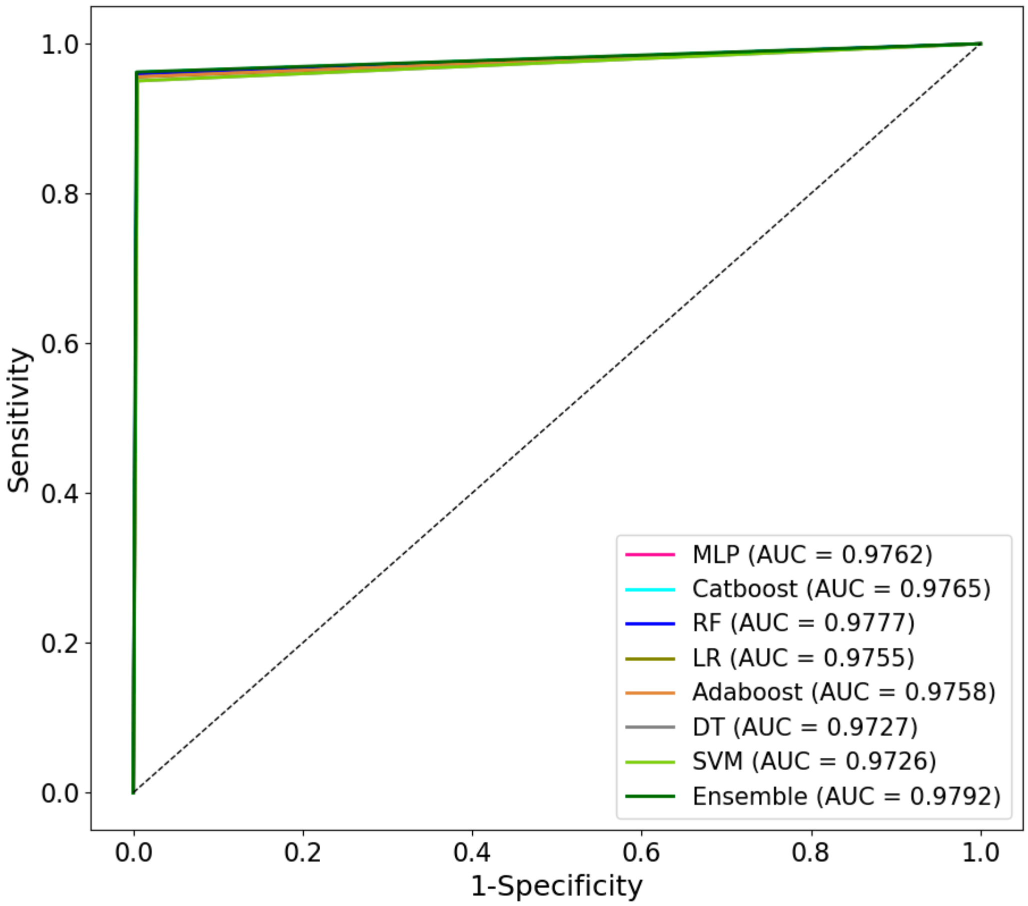

Figure S2. ROC curves of different classification methods on the GISAID testing dataset.

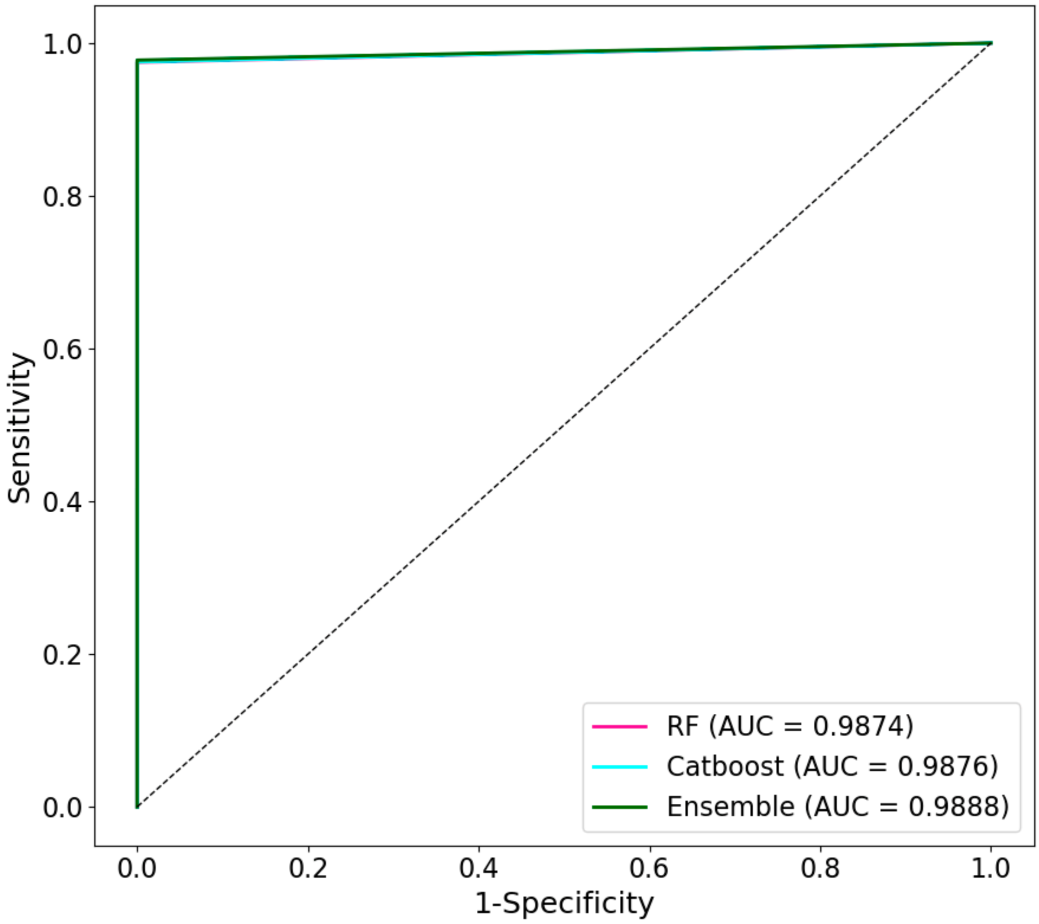

Figure S3. ROC curves of different classification methods on the Pangolin testing dataset.
